# Health Promoting Properties of Bee Royal Jelly: Food of the Queens

**DOI:** 10.3390/nu13020543

**Published:** 2021-02-07

**Authors:** Nicolas Collazo, Maria Carpena, Bernabe Nuñez-Estevez, Paz Otero, Jesus Simal-Gandara, Miguel A. Prieto

**Affiliations:** 1 Nutrition and Bromatology Group, Department of Analytical and Food Chemistry, Faculty of Food Science and Technology, Ourense Campus, University of Vigo, E32004 Ourense, Spain; nicolascollazojimenez@gmail.com (N.C.); maria.carpena.rodriguez@uvigo.es (M.C.); bernabenunez16@gmail.com (B.N.-E.); paz.otero@uvigo.es (P.O.); 2 Centro de Investigação de Montanha (CIMO), Instituto Politécnico de Bragança, Campus de Santa Apolonia, 5300-253 Bragança, Portugal

**Keywords:** royal jelly, biological properties, health applications, 10-hydroxy-2-decenoic acid, main proteins of royal jelly, authenticity

## Abstract

Royal jelly (RJ) demand is growing every year and so is the market for functional foods in general. RJ is formed by different substances, mainly carbohydrates, proteins, and lipids, but also vitamins, minerals, and phenolic or volatile compounds in lower proportion. Major royal jelly proteins (MRJP) are, together with 10-hydroxy-2-decenoic acid (10-HDA), key substances of RJ due to their different biological properties. In particular, 10-HDA is a unique substance in this product. RJ has been historically employed as health enhancer and is still very relevant in China due to the traditional medicine and the apitherapy. Nowadays, it is mainly consumed as a functional food or is found in supplements and other formulations for its health-beneficial properties. Within these properites, anti-lipidemic, antioxidant, antiproliferative, antimicrobial, neuroprotective, anti-inflammatory, immunomodulatory, antiaging, and estrogenic activities have been reported for RJ or its specific components. This manuscript is aimed at reviewing the current knowledge on RJ components, their assessment in terms of authenticity, their biological activities, and related health applications.

## 1. Introduction

**Figure 1:**
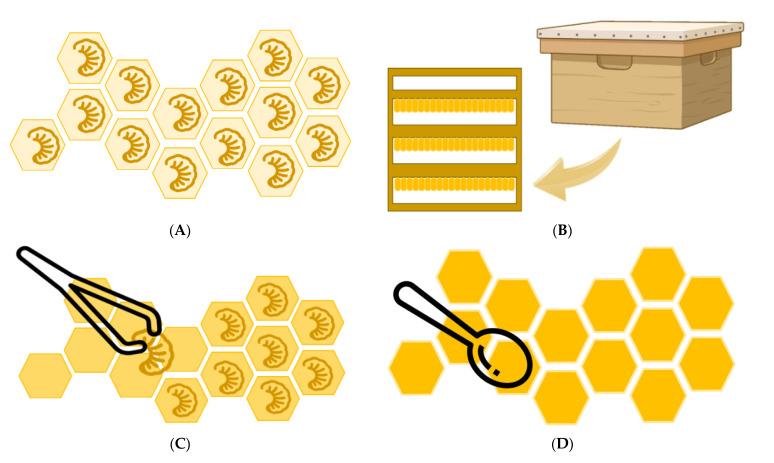
Royal jelly (RJ) production in royal cells inside a hive. (**A**) RJ production in queen cell base with worker bee larvae inside. (**B**) Hive for breeding honeybees and queen bees for RJ production. (**C**) Removal of worker larvae from royal cells. (**D**) Collection of RJ from the cells.

Royal jelly (RJ) is a yellowish-white, creamy, acidic secretion from the mandibular and hypopharyngeal glands of young worker bees of the *Apis mellifera* species [1,2,3]. Although the literature usually states that all bee larvae, workers, and queens are fed with jelly for the first three days after hatching, and only the queen larvae continue to be fed with RJ throughout their development, a clarification must be stated. During the first 3 days, nurse bees provide two different larval foods for queen and workers—the former is called the RJ and the latter is the worker jelly. Nurse bees provide a larger quantity and higher quality of food to the larvae in the queen cells, which causes the larvae to synthesize large amounts of juvenile hormone at 3 days old, thus leading to the development of the queen bee. The unique composition of RJ causes changes in gene expression, allowing, for example, full ovarian development to continue in the queen. Thanks to the RJ, the queen can live for up to five years, while the workers usually live about 45 days, and can lay about 2500 eggs a day [2,4,5,6,7,8]. The main method of production of RJ consists in the grafting of artificial larvae. Worker bee larvae, 12 to 18 h after hatching, are transferred using a grafting pen, to artificial queen cell bases to induce colony worker bees to produce RJ to feed the larvae. After 68–72 h (3 days), the larvae are removed with tweezers from the bases and the RJ is collected and transferred to a RJ bottle for storage. Larval grafting is a time-consuming and labor-intensive step, so a new method for the production of RJ that does not require larval grafting was described in recent years [5]. Figure 1 shows the production process of RJ in queen cell bases. RJ has a complex composition of water, proteins, carbohydrates, fatty acids and lipids, minerals and small amounts of vitamins, free amino acids, and volatile compounds [7,9,10,11,12]. However, it is difficult to gather data collected by several authors because of the different locations of the RJ samples and their inhomogeneous nature [10]. The most characteristic compound of the RJ is *trans*-10-hydroxy-2-decenoic acid (10-HDA), which is a unique active substance present in RJ [8,13,14]. In addition, proteins are the most abundant compounds in RJ and are usually called major royal jelly proteins (MRJPs) [11].

There are no official data on the RJ market, but China is undoubtedly recognized as the largest producer of RJ [10]. It produces around 3500 tons/year, which accounts for more than 60% of the world’s production, and is exported almost entirely to the United States, Europe, and Japan [7,15,16]. Other countries such as Korea, Japan, and Taiwan are also major producers and exporters of RJ. In Europe, more RJ is produced in Eastern European countries than in Western Europe [7,10]. The lack of quality criteria and control of authenticity and geographical origin deters beekeepers from expanding their businesses, causing the industry to grow very slowly, and these growths are Chinese imports that cover local demand in many countries with highly competitive prices [12,17]. There are currently no European or international standards for bee products other than honey, but many countries have established their own national standards. The first country that set the criteria for RJ was Argentina in 1979, followed by Bulgaria in 1984, Poland in 1996, Turkey in 2000, Brazil in 2001, Serbia in 2003, Switzerland in 2005, Japan and China in 2008, India in 2012, and Korea in 2014 [18].

Since ancient times, RJ has been used in traditional medicine, especially in Asian apitherapy and in ancient Egypt [11]. However, over the last few years, the interest of consumers and food industry in healthy natural products has gradually increased in order to promote health and reduce disease [8]. Due to its excellent biological properties, RJ is one of the most attractive functional foods, being used as a dietary supplement and in various industries, such as pharmaceutical, food, and cosmetics [7,19]. Several studies reported RJ biological activities such as antimicrobial [1,20], antitumor [21], hepatoprotective [22], immunomodulatory and anti-hypercholesterolemic [23], antioxidant [24], and antidiabetic [24].

The purpose of this study is to carry out a review of the main constituents of RJ and their biological and health promoting properties. In addition, a wide variety of applications in the nutraceutical and cosmetic industries are addressed in the manuscript together with a brief revision of the main issues associated with RJ authentication.

## 2. Composition of Royal Jelly

RJ is an acidic colloid, whose pH usually ranges between 3.6 and 4.2, although other authors have expanded this range between 3.4 and 4.5 [25,26]. Water constitutes the major component (50–70%), followed by proteins (9–18%), carbohydrates (7–18%), lipids (3–8%), trace minerals (0.8–3%), vitamins, phenols, and amino acids [2,7,10,15]. Hence, the different values obtained by different authors fluctuate within a certain range. This is due to the heterogeneous nature of RJ, and the different samples taken from different locations at different times of production. However, the most variable parameters are sugars and lipids [10]. Depending on multiple factors (season, location, botanical origin, among others), RJ sugar content significantly varies from sample to sample. French RJ has lower contents of sucrose and erlose [12,25]. Lipid content is affected by RJ type as well. Italian samples have a higher lipid content than commercial samples. The total amount of fatty acids is not influenced by the RJ type of sample, as is the total amount of sterols. However, there is a high degree of variability according to the predominant sterol type between Italian and commercial samples [27]. Environmental conditions significantly affect the chemical composition. During the rainy season, water, and carbohydrates reach maximum levels, while the highest level of lipids reaches the maximum in the dry season. Protein content was lightly altered throughout the year, while mineral content and pH value were constant [28]. The main composition of RJ and its different biological activities are described below and collected in Table 1.

### 2.1. Carbohydrates

The sugar portion accounts for between 7 and 18% of the total in fresh RJ and about 30% of the dry matter [10,49]. Overall, the total fructose and glucose content accounts for 90% of total sugars. The average concentrations are: fructose (2.3–7.6%, mean 4.9%) and glucose (2.9–8.1%, mean 5.5%) [12]. Whatever the RJ’s origin, fructose and glucose contents are in the range of 2.3–7.8% and 3.4–8.2%, respectively [75,76,77].

On the contrary, large differences are observed in the content of minor sugars, playing a providential role in the control of the origin and authenticity of the product [10,49]. Sucrose is always present but often in variable concentrations: <0.1–2.1%, mean 1.0% [77], 0.8–3.6%, mean 1.87% [75], 0.0–1.6%, mean 0.6% [17]. Sucrose and erlose contents in French RJ are between 0.0 and 1.8% (mean 0.5%) and 0.0–0.4% (mean 0.1%), respectively, as they reach 3.9 and 2.0% in some commercial samples and 7.7 and 1.7% in RJ produced by sugarcane feeding. Maltose and maltotriose contents are 0.0–1.0% (mean 0.3%) and 0.0–0.2% (mean 0.02%) in French RJ, and they attain levels of 2.6 and 0.4% in commercial samples, and by feeding bees with starch hydrolysate syrups can reach 5.5% and 1.7%. These differences in percentages are due to the effects of different types of feeding on bees [12]. In addition, more minor sugars, such as galactose, mannitol, maltulose, turanose, trehalose, palatinose, isomaltose, gentiobiose, and melezitose, and gluconic acid, a product derived from glucose oxidation, have also been detected [12,76].

It is thought that sugars contribute to RJ’s epigenetic effects, representing a phagostimulant that works through the insulin/insulin-like signaling cascades and the mTOR nutrient detection pathway to derive larval development by increasing amounts of food ingested and increasing intake of nutrients needed for queen development [4,25].

### 2.2. Proteins

Proteins account for >50% of dry weight and the so-called major proteins of royal jelly (MRJP) constituted about 80–90% of soluble RJ proteins [25,30,78,79]. MRJP are recognized as the main factor involved in specific physiological actions of RJ [42]. The nine members within this family (MRJP 1–9) are heavily homologous and have theoretical molecular masses of 49–87 kDa [5,80]. They have been named according to their molecular weight or order of its discovery [26]. Since the first five MRJPs account for between 82 and 90% of MRJPs, they are thought to have an important nutritional role and they are the most important nitrogen reserve. MRJP 6–8 have no apparent nutritional function [20,25,80].

MRJP1 is a weak acidic glycoprotein which comprises about 48% of all proteins in RJ [15,30,81]. It is found in either monomeric or oligomeric form. The monomeric form, also known as apalbumin I or royalactin, is a 55 kDa protein. The oligomer, known as apisin, has been estimated to be between 280 and 420 kDa [70,82,83,84,85]. Tian et al., (2018) reported that apisin adopts a H-like structure of 254 kDa formed by four MRJP1, four apisimin, and eight 24-methylenecholesterol at neutral pH. Apisimin is a serine-valine-rich 40 kDa peptide only found in honeybees. Apisin could be used to determine the quality of RJ [86,87], although its physiological function is still unclear [82]. Royalactin induces the differentiation of honeybee larvae into queens [88].

MRJPs 2–5 are also glycoproteins of 49, 60–70, 60, and 80 kDa molecular weight, respectively [42,78]. MRJP2 is a lightly basic protein, whilst MRJP 3–5 are almost neutral and MRJP7 is an acidic protein. MRJP8 and 9 are both rare in RJ, but could be detected in the venom gland [5,80,82]. Most of the MRJPs contain high amounts of the 10 essential amino acids in honeybees: Arg, His, Ile, Leu, Lys, Met, Phe, Thr, Trp, Val. The highest essential amino acids content is seen in MRJP5 (51.4%), MRJP1 (48%), and MRJP2 (47%) [78,80].

RJ also contains other proteins than MRJPs, but in a much smaller amount. Royalisin is an antimicrobial protein of the insect defensine family, active against a large spectrum of Gram+ and Gram- bacteria and fungi [48]. Jelleins are four peptides derived from the C-terminus of MRJP1. Jelleins I, II and III have antimicrobial activity against Gram+ and Gram- bacteria and yeasts, whereas jellein-IV has no antimicrobial effect [1,26,49]. Jelleins and royalisin together provide a wide-spectrum antimicrobial protection of the RJ [1,20].

Free amino acids (FAA) are another important component of the RJ. The average content of FAA in fresh RJ is 9.21 mg/g [89]. The major L-series FAAs are proline (2.4–5.4 mg/g), lysine (0.6–2.2), glutamate (0.5–0.9), ß-alanine (0.3–0.5), phenylalanine (0.2–0.6), aspartate (0.2–0.5) and serine (0.1–0.3). The D-Series FAA concentration was below the method detection limit (0.1 mg/g GR) [90]. Using the LC/MS method, lysine was the most prominent FAA (62.43 mg/100 g) ahead of proline (58.76 mg/100 g) [91,92]. Proline is thought to protect membranes and proteins from stress conditions and also act as an antioxidant. Cysteine is involved in the synthesis of glutathione, an effective cellular antioxidant [92].

### 2.3. Lipids

The lipid´s fraction appears in variable concentrations—3–8% in fresh RJ and 7–19% in dry RJ [2,10]. The majority of lipids (80–90%) are free fatty acids, with few being esterified. Phenols (4–10%), waxes (5–6%), steroids (3–4%), and phospholipids (0.4–0.8%) have also been reported [7,15,59,62]. Around 80–90% of the fatty acid fraction has a fairly unusual structure in nature, consisting of mono- and dihydroxy- acids and dicarboxylic acids with 8 and 10 carbon atoms in the chain. Most of the biological properties are associated with these lipids [2,10]. 10-HDA constitutes about 70% of total RJ lipids and more than 50% of free fatty acids. The precursor of 10-HDA, 10-hydroxydecanoic acid (HDAA), constitutes 17% of free fatty acids. Together, 10-HDA and 10-HDAA make up 60–80% of the fatty acids identified [27,59]. HDA and HDAA are specific components of RJ [62]. 10-HDA is known for having many pharmacological and health effects [7]. Other dominant fatty acids are 8-hydroxy octanoic acid, 3-hydroxydecanoic acid, 3,10-dihydroxydecanoic acid, and 9-hydroxy-2-decenoic acid [59].

RJ contains sterols in minor amounts, originating from plant sources. 24-methylene cholesterol constitutes 49–58% of RJ sterols [15,60]. Two 4-desmethylsterols, β-sitosterol and Δ5-avenasterol, are the next major sterols, whereas cholesterol, desmosterol, campesterol, stigmasterol and Δ7-avenasterol are found at lower concentrations, less than 0.1 mg/g of total lipids. [59,70].

### 2.4. Vitamins

Pantothenic acid (vitamin B5) and niacin (B3 or PP) are the most abundant vitamins in RJ: 52.8 and 42.42 mg/100 g, respectively. RJ also contains small quantities of various B-complex vitamins (B1, B2, B6, B8, B9, and B12), vitamin C, D, E, and A [18,25,49,93,94].

Pantothenic acid is recognized for being a lifespan-extending agent and for its ability to reduce stress levels and reverse hair aging. Therefore, RJ has been used as a treatment for hair. Biotin promotes keratin production [18,25].

### 2.5. Minerals

Ash content constitute 0.8–3% of RJ fresh matter and 2–5% of RJ dry matter [9,10]. It contains not only different minerals—K^+^, P^3−^, S^2−^, Na^+^, Ca^2+^, Al^3+^, Mg^2+^, Zn^2+^, Fe^2+^, Cu^+^, and Mn^2+^—but also it contains trace elements such as Ni, Cr, Sn, W, Sb, Bi and Ti [15,20,25]. The main element is potassium (2462–3120 mg/kg), followed by phosphorus (1940–2350 mg/kg), sulfur (1420–1154 mg/kg), calcium (145–113 mg/kg), magnesium (264–312 mg/kg) and sodium (106–142 mg/kg) [95]. The functions of potassium in the body are varied. It regulates fluid balance, controls the electrical activity of the heart and other muscles, reduces blood pressure, decreases the risk of stroke, and preserves bone mineral density [18]. Zn, Fe, Cu, Al, and Mn are the most abundant trace elements in RJ. Trace elements play a key role in the biomedical activities associated with RJ, and these elements have a multitude of known and unknown biological functions [7,95,96].

Depending on the botanical origin and the type of soil, trace and mineral concentrations widely vary between different samples of honey and pollen [97]. However, mineral and trace concentrations from different RJ samples do not show significant variability despite having different botanical and geographical origins. This fact highlights the homeostatic adjustment of trace and mineral element concentrations, produced in the endocrine glands of nurse bees. RJ shows the same homeostatic adjustment as mammalian and human breast milk [95,96].

### 2.6. Phenolic and Volatile Compounds

There are few references to volatile compounds (VC) in RJ, being the least studied and identified components of it [13,65,98,99,100]. However, the mixture of these various VC directly affects the taste and quality odor of the RJ [98,99]. There are great differences between the results obtained by different authors in the composition of the volatile fraction. This is because VC can be influenced by different factors, such as honeybee species, harvesting time, geographical origin, storage methods, or processing technologies [98].

Isidorov et al. (2009) determined by headspace solid-phase microextraction/gas chromatography-mass spectrometry (HS-SPME/GC–MS) a list of 25 different compounds in the volatile fraction of RJ from two *Apis mellifera carnica* colonies in Poland. The most abundant compounds were carbonyls (37–43%), with 2-heptanone at the head representing about 20% of the total. The remaining carbonyl compounds identified were, in decreasing order: acetone > 2-nonanone > benzaldehyde > 2-butanone > 2-pentanone and hexanal [13,101]. It has been reported the repellent effect of 2-heptanone, which can be directed against the destructive Varroa mite, an ectoparasite of the honeybee *Apis mellifera* L. It can also increase the repellent action of octanoic acid, 7% of the total volatile fraction. This compound can interfere with the process of cellular invasion by the mite [13,65]. In addition, the volatile phenolic compounds phenol, o-guiacol, and methyl salicylate are another significant group (10–20%). These compounds, together with benzoic acid (2.5–3.6%), possess antibacterial properties. The main constituents of VC (47%) are protective components whose activities are directed against both parasites and microorganisms [13]. Many types of polyphenolic compounds, with high antioxidant activity, are present in RJ [15,25]. Ferulic acid is the only phenolic acid found, comprising more than 68% of the total amount of polyphenols. In lower percentages, compounds belonging to flavanons, flavones, flavonols, and isoflavonoids have been found [102].

Also using HS-SPME/GC–MS technique, Zhao et al. (2016) determined 40 VC in 10 different RJ samples. Esters were the most abundant VC accounting for 25%, followed by aldehydes (17.5%), ketones (15%), acids (10%) and alcohols (10%). There was a significant variation of different classes of compounds in each RJ sample type. However, 2-nonanone and acetic acid were both detected in all samples, whereas toluene, benzaldehyde, octanoic acid, 2-pentanone, benzoic acid, and methyl ester were found in eight to nine samples. Acetic acid, benzoic acid methyl ester, hexanoic acid and octanoic acid contribute much more to RJ flavor and can be used for differentiating different RJ [98].

## 3. Royal Jelly Authenticity and Adulteration

**Figure 2:**
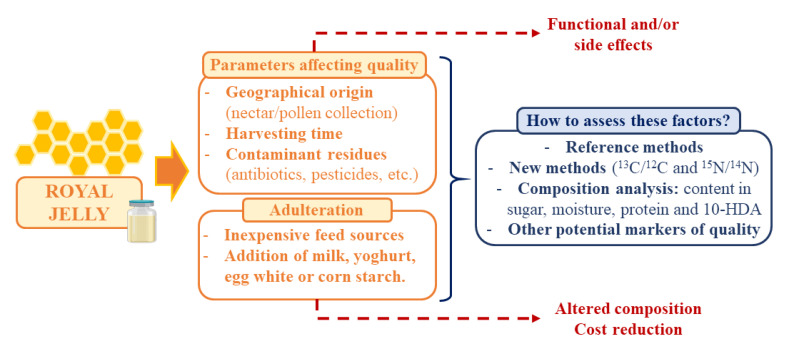
Summary of the main parameters affecting RJ quality, adulteration techniques and measures to assess these factors and guarantee the final quality of the product.

These days, RJ is considered as an attractive functional food but also an expensive product that can be submitted to adulteration as means to reduce production costs. In addition, other parameters can affect the final quality of RJ, such as the influence of geographical origin, harvesting time, or the presence of contaminant residues [7,103]. Furthermore, the use of reference methods to analyze the composition of RJ and the assessment of these factors is essential to guarantee the quality of the product and its expected physicochemical and organoleptic properties (Figure 2).

Naturally, RJ is produced by the transformation of nectar and pollen collected by bees [12]. In large production systems, the most common sugar sources for bee-feeding are sugar syrups or honey and yeast powder or pollen as protein sources [103]. So, the use of inexpensive feed sources is a tempting option for reducing costs, which has been previously reported. The studies on this area are focused on establishing new methods for evaluating adulteration and authenticity, such as the ^13^C/^12^C and ^15^N/^14^N stable isotope ratios mass spectrometer [104]. The most common criteria for assessing RJ quality and authenticity is sugar, moisture, protein, and 10-HDA [12]. In addition, other studies have highlighted certain amino acids, amines, carbohydrates, and vitamins as potential markers [103]. Among all of them, 10-HDA is the main marker for RJ quality and authenticity [10]. However, the measure of 10-HDA content cannot be considered as a good freshness criterion [105]. Proteins, despite their importance and quantity, are also not good indicators of freshness [106]. Moreover, adulteration has been performed by adding milk, yoghurt, egg white, or corn starch [7,106]. For instance, RJ has been adulterated with powdered milk, since the taste characteristics are similar to pure RJ [107]. Melamine is a triazine ring with three amino groups commonly used for plastics or resin production that has been associated to possible incidence of nephrolithiasis and related deaths [7,108]. This compound has been also found in RJ by hydrophilic interaction chromatography/tandem mass spectrometry. It was added to apparently increase the protein content of RJ over its real value [108].

Alternatively, bees’ diets are linked to the environment, so RJ geographical origin will determine its final composition. A recent study showed that environment (e.g., coastal or inland areas) could modify RJ composition. Furthermore, the distinct composition in the sugar content was apparently correlated with radical free radical scavenging activity [19]. In addition, geographical origin can be inferred from pollen analysis [7]. Likewise, harvesting time will also affect RJ characteristics. A recent study proposed that VC could be used for distinguishing RJ harvested in flowering seasons and discriminating different pollen and nectar plants [98].

At last, exposure to different substances can modify RJ production and characteristics. Veterinary drugs are one common class of residues produced as a result of their use for prevention of specific diseases. Chloramphenicol, nitroimidazole, sulphonamides, fluoroquinolone, or tetracycline are a few examples of the antibiotics that can be retained in RJ [5,109,110]. Another important source of contaminants is pesticides. The repeated use of these products on the environment has been demonstrated to have functional and behavioral effects on bees, but they also entail other side effects. In a recent study, carbendazim treatment during larval stage caused the down regulation of the expression of MRJP [111]. Other authors evaluated the effects of exposing colonies to a multi-pesticide (nine compounds) pollen treatment and found that RJ production was reduced together with some target compounds such as MRJP or 10-HDA [112]. Similarly, neonicotinoid insecticides are widely used in the world and due to their translocation ability, they can pass throughout the plants to pollen or nectar and reach bees [113]. Several studies have demonstrated the presence of these residues in RJ by performing ultra- or high-performance liquid chromatography coupled to mass spectrometry [113,114,115]. Overall, the adulteration and authenticity of RJ needs to be further studied to set the optimal composition standards and guarantee the quality and authenticity of the product to avoid food fraud and the loss of its essential characteristics and properties.

## 4. Biological and Health Promoting Properties

RJ is one of the most popular functional foods. This product was commercialized, principally, for its dietetic and cosmetic activity. However, RJ has been demonstrated to have many pharmacological activities which have the potential to prevent or combat numerous diseases [7]. Recently, diverse works have been performed on RJ’s bioactivities, giving a broader vision of how RJ can contribute to the development of drugs and human health.

### 4.1. Anti-Lipidemic Activity: Lipid Metabolism

**Figure 3:**
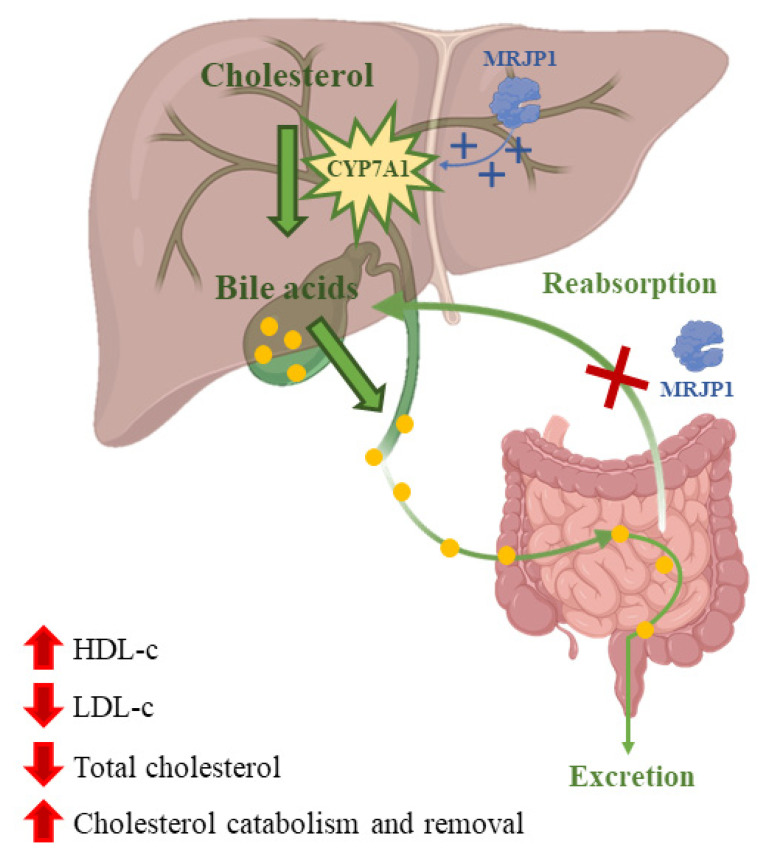
Cholesterol is converted into bile acids catalyzed by the enzyme cholesterol 7-α-hydroxylase (CYP7A1). Bile acids are then excreted or reabsorbed at the jejunum. MRJP1 upregulates the expression of CYP7A1 increasing bile acids synthesis and their excretion. In addition, MRJP1 can inhibit cholesterol and bile acid reabsorption.

Nowadays, dyslipidemia is a high-risk factor for cardiovascular diseases that gets worse due to bad eating habits. Atherosclerotic cardiovascular disease is produced by low levels of high-density lipoprotein cholesterol (HDL-C) and high levels of triglycerides and low-density lipoprotein cholesterol (LDL-C) in the plasma [116]. Many studies have been done on how RJ affects lipids concentration in blood. A meta-analysis of those studies showed that treatments with RJ could reduce total cholesterol in the blood and increase HDL-C levels [117]. These changes in the concentration of blood lipids could be caused by the inhibition of the absorption of cholesterol in the jejunum by the MRJP. Moreover, MRJP1 can also block the reabsorption of bile acids [33]. Furthermore, RJ could produce the upregulation of cholesterol 7-α-hydroxylase (CYP7A1) that would increase the activity of hepatic receptors for the synthesis of very low-density lipoproteins, which are precursors of LDL-C (Figure 3) [118]. RJ has been suggested as a hypocholesterolemic agent since total cholesterol and LDL-C levels were reduced with an intake treatment of nine capsules (350 mg/capsule) of RJ or placebo/day, respectively, for three months [119]. Another study found that dietary RJ was able to suppress high fat diet-induced accumulation of white adipose tissue and liver and to increase brown adipose tissue thermogenic capacity in mice without modifying food and energy levels [120]. However, previous works have stated that the effects of RJ on lipid profile are contradictory [121].

### 4.2. Antioxidant Activity: Oxidative Stress

Oxidative stress produced by the liberation of reactive oxygen species (ROS) in the body is related to some pathological processes [122]. In several studies, the multiple benefits of the antioxidant activity of specific compounds against these pathologies have been demonstrated. At the beginning of this century, it was demonstrated that RJ has antioxidant activity. Moreover, it was discovered that this activity came from its proteins (MRJP 1–9) and peptides [94,123]. Since then, numerous studies have been carried out on how the antioxidant capacity of RJ can help treating different pathologies. For instance, a recent study evaluated the antioxidant capacity of RJ by 2,2-diphenyl-1-picrylhydrazyl (DPPH) free radical scavenging activity and found that it ranged from 102 to 354 mg/mL and that there was no apparent link between antioxidant activity and geographical origin or phytochemicals [19].

Recently, the in vivo hepatoprotective activity of RJ against nonalcoholic fatty liver disease (NAFLD) was tested. NAFLD is the liver disease with greatest incidence in the world, with a prevalence of 25% in the population [124]. Factors such as age, postmenopausal status, and obesity increase the risk [125]. Moreover, NAFLD can lead to necrotic inflammation, fibrosis, and cirrhosis, and increases the risk of suffering other illnesses such hepatocellular carcinoma or cardiovascular diseases [126]. The pathogenesis of NAFLD is closely related to oxidative stress in hepatic cells [127]. Mice previously ovariectomized were treated with different doses of RJ (150, 300 and 450 mg/kg) intragastrical. In the control, an improvement in the oxidative stress level caused by an increment of the lipid peroxidation in the liver and the rise of nitric oxidase and malondialdehyde levels was observed. The mice treated with RJ had reduced oxidative stress thanks to an increase in hepatic antioxidant enzymes. Furthermore, RJ possesses compounds with antioxidant activity that could help to control the oxidative stress exerting hepatoprotective activity in ovariectomized mice [128]. Alternatively, another experiment was performed to observe the antioxidant activity of RJ. Rats with induced nephrotoxicity by cadmium administration were pretreated with different doses of RJ. The administration of cadmium produced a kidney dysfunction caused by an increase in ROS and reactive nitrogen species (RNS) resulting in cellular damage in the control group. Groups that were pretreated with RJ where able to restore the oxidative stress, decreasing the NO and lipid peroxidation to normal levels [129].

### 4.3. Antiproliferative Activity

Several strategies can combat cancer, but most of them also have some detrimental effect on the health of patients. One strategy to combat the growth of tumor cells without so many negative effects is to decrease their replication by natural antiproliferative compounds. RJ was demonstrated to have antitumor activity against Lewis lung carcinoma and colorectal adenocarcinoma cells [130,131]. To determine if RJ compounds have antiproliferative activity against cancer cells, six different types of RJ were tested against SH-SY5Y human neuroblastoma cells in vitro. For the study, the hydrophilic and the lipophilic compounds of the RJ were separated and administrated to the cell cultures in different concentrations (250, 750 and 1250 μg/mL). The results were similar for the six types of RJ; all of them presented a high antiproliferative effect in the cell cultures treated with the lipophilic extract. This antiproliferative activity could be linked to the 10-HDA that in other studies presented differentiating and neurotrophic activity on murine neuronal cells [132]. The connection between 10-HDA and RJ has been proposed previously, e.g., in 1960, it was found that it inhibited the development of transplantable AKR leukemia and different lines of ascitic tumors in mice [133]. 10-HDA has also shown an antiproliferative effect against fibroblast-like synoviocytes of rheumatoid arthritis (RA) patients by PI3K–AKT pathway. 10-HDA acts as HADACIs (histone deacetylase inhibitors), which have been considered as drugs with strong anti-inflammatory activity, related to the down-regulation of PI3K and the phosphorylation of AKT [134]. Another study assessed the inhibition of vascular endothelial growth factor (VEGF) induced angiogenesis by avoiding the proliferation of human umbilical vein endothelial cells [57].

### 4.4. Antimicrobial Activity

The search for new antibiotics to counteract the high level of antibiotic resistance is a priority to control different pathogens. In this search, antimicrobial peptides (AMPs) appear to be a natural alternative to conventional antibiotics [135]. The positive charge of these compounds allow them to interact with cell membranes that are negatively charged [136]. The antimicrobial capacity of AMPs comes from its ability to disrupt bacterial cells, causing the death of the bacteria. There are four theories (barrel-stave model, carpet-like model, toroidal pore model and aggregate channel model) of disruption that can explain this antibacterial activity but the exact mechanism of action is still unknown [137]. Compounds of RJ like jelleins, royalisin, MRJPs, royalactin, and apisimin could be classified as AMPs [20]. Moreover, there are other compounds of RJ with antimicrobial activity out of the AMPs, such as 10-HDA, which exhibits growth-inhibitory activity against different bacteria [138].

Since the first time that RJ was studied for its antibacterial activity [139], several studies have been performed (Table 2). These studies demonstrated the high antibacterial activity against Gram-positive bacteria and, in a minor way, against Gram-negative bacteria of RJ and its principal compounds. Alternatively, in a recent study, the RJ was tested as an anti-biofilm agent. Formation of biofilms is a big problem in alimentary industries for the contamination of food and drinks and the possibility to infect consumers. RJ was tested against *Listeria monocytogenes*, which is usually present in food biofilms, and produced 2549 infections in 2018 in Europe [140,141]. In this study, *L. monocytogenes* cultures were treated with different concentrations (0.33–41.67 mg/mL). The results show that the minimum inhibitory concentration (MIC)_90_ was 23.85 mg/mL. This result confirms the possibility of using RJ as an antibiofilm agent to reduce the risk of listeriosis contamination [142].

### 4.5. Neuroprotective Effect

The aging of the population in developed countries is increasing the number of neurodegenerative disorders. Alzheimer’s disease (AD) and Parkinson´s disease are two of the most important neurodegenerative diseases in developed countries due to their prevalence in the old population and their specific symptoms [148,149]. Therefore, a search for new therapies is required to mitigate the repercussions in the population. RJ has demonstrated its neuroprotective effects [150,151,152,153]. Recently, a study about the capacity of RJ to reduce cadmium-induced neuronal damage in vivo was carried out. Cadmium exposition produces impaired neurodevelopment associated with AD [154]. The study was completed in mice divided into three control groups (control, cadmium control and RJ control) and a RJ-cadmium group. The RJ-cadmium group was treated with 85 mg/kg of RJ orally administrated and with 6.5 mg/kg CdCl_2_ by intraperitoneal injection. The results show neuroprotective activity. This protection could be produced by the RJ antioxidant activity, reducing the lipid peroxidation and NO levels. Moreover, RJ administration restored the activity of antioxidant enzymes (GSH-Px1, GSH-R, SOD2, and CAT) that were depleted by the Cd-intoxication. Furthermore, the treatment was able to upregulate the Nrf2 that regulates the gene expression of antioxidant enzymes [155]. Additionally, TNF-α and IL-1β levels were decreased, reducing brain inflammation. In addition, anti-apoptotic activity by the downregulation of Bax and caspase 3 and upregulation of Bcl-2 was found, reducing the neuronal damage [156].

One predisposing factor in woman of old age is menopause. Menopausal hormonal alteration produces an increase in the risk of suffering neurodegenerative diseases such as AD and diseases related to autonomic nervous system dysfunction [157,158]. Recently, an in vivo study was performed to figure out if RJ could possess beneficial properties against the adverse neurological effects of menopause. The study was performed in ovariectomized rabbits fed with a high-cholesterol diet. After the ovariectomization, 400 mg/kg of RJ was orally administrated. The results of the study show beneficial neurological effects against postmenopausal neurological disorders. This neuroprotective effect could be produced by RJ components such as 10-hydroxy succinic acid, trans-2-ylenic acid, and 10-hydroxy-2-olenoic acid, which have estrogen-like effects. Moreover, RJ improved the estradiol and progesterone levels that can attenuate mild cognitive impairments [159]. In previous sections the capacity of RJ to reduce cholesterol levels was displayed. Therefore, this reduction in blood lipids could also produce neuroprotective effects by means of the reduction in amyloid-beta concentration in the brain, which undergoes upregulation with high cholesterol levels. Furthermore, RJ reduces the expression of β-site APP cleaving enzyme, which is related to high levels of amyloid-beta [153,160]. Additionally, the presence of sterols and some fatty acids in RJ with estrogenic activity can activate the estrogen receptor β in the brain due to their capacity to cross the blood–brain barrier due to their low polarity [60]. RJ also can improve the cholinergic system and antioxidant capacities, producing an enhancement of the autonomic nervous system [153].

### 4.6. Anti-Inflammatory Activity

Inflammation is one of the first responses of the body against infection or injury and produces the beginning of the immunological process. Inflammation is a normal process of the innate immune response, but if it is not well regulated it can produce tissue damage, and is derived in some pathological disorders [161]. RJ has shown anti-inflammatory activity in different diseases related to an abnormal inflammation [11,41,61,162]. To understand better the mechanism of this activity, in a recent study, murine microglial cell line BV-2 was cultivated with RJ and later cultivated with lipopolysaccharides that stimulate the inflammatory response [163]. The results show that cells treated with RJ presented a better response to the inflammation. This protective effect could be caused by the inhibition of the transcription of TNF-α, IL-1β and IL-6 cytokines that are pro-inflammatory.

Furthermore, RJ could inhibit the expression of the pro-inflammatory protein COX-2. Moreover, in this study, RJ also presented antioxidant effects, reducing NO and ROS levels in the cells. In addition, RJ presented an inhibitory effect on the production of inflammatory mediators through JNK, p38 and NF-*k*B pathways [163]. Alternatively, a recent study performed in asymptomatic overweight adults showed anti-inflammatory benefits in the patients. The patients were treated with 333 mg per day by oral ingestion of capsules for 8 weeks. The RJ administration produced a decrease in the inflammatory marker CRP and an increase in anti-inflammatory adiponectin, and IL-6 cytokine levels were reduced. Furthermore, RJ increased the expression of adiponectin receptor 1 as well as the adiponectin that could be linked to the increase in the peroxisome proliferator-activated receptor-*α*, AMP-activated protein kinase (pAMPK), and peroxisome proliferator-activated receptor gamma coactivator 1-*α* [164,165].

### 4.7. Additional Effects

Aging affects the muscles and bones of the old population. Therefore, new treatments and functional food development are very important to improve the health and quality of life of elderly people. RJ presented an improvement in the bone quality of ovariectomized rats by a reduction in collagen crosslink (pyridinoline and deoxypyridinoline) and stimulation of the expression of collagen-modifying enzymes. These effects produced posttranslational modifications in type I collagen [166]. To find the compounds involved and the mechanism of this activity, a study in vitro with 10-hydroxy-2-decenoic acid (10H2DA) was recently performed. In the study, ovariectomized mice were orally treated with RJ (1 g/kg of body weight) and 10H2DA (40 mg/kg of body weight). The results show a suppression of osteoclastogenesis decreasing bone resorption [167]. This effect could be caused by the inhibition of NF-kB signaling through the FFAR4 receptor [168]. Alternatively, RJ demonstrated positives effects in the function and conservation of the skeletal muscle [169,170,171,172]. To improve this effect, RJ treated with proteases was tested against denervation-induced skeletal muscle atrophy. In this study, mice were treated with protease-treated RJ (pRJ) orally for 3 weeks. Afterwards, a fragment of the sciatic nerve was cut and excised. After six days, a section of muscle was collected. The results show that the treatment improved the decrement of myofiber size after denervation. Moreover, pRJ produced an increment in the expression of regeneration-related genes (IGF-1 and IGFR) [173].

## 5. Royal Jelly Health Applications

### 5.1. Nutraceutical Industry

RJ has been historically employed as a health enhancer and is still very relevant in China due to traditional medicine and apitherapy. The first chemical analysis of RJ was carried out in 1852 by the American Reverend Langstroth; nevertheless, his analyses did not guarantee reliable information. During the 1850s, Langstroth proposed the commercialization of RJ as a solution in areas where honey was not produced, and its use as a functional product was enhanced from 1860 due to the discovery of its properties [20]. From then on, RJ has been produced in large scale for commercial purposes and marketed as capsules, tablets, in ampoules, etc. China produces 90% of the global production (4000 tons of RJ per year) and mainly exports to Japan, Europe, and the United States. The increase in the production of RJ during the last 4 decades is mainly due to the development and optimization of production techniques and the development of genetic selection of Italian bees so that they are capable of producing up to a 10 times greater amount of RJ than the bees not selected [15]. RJ industry applications and some of their benefits to promote human health are described below and collected in Table 3.

#### 5.1.1. Functional Food

Reliance on foods with health benefits has been encouraged by socioeconomic changes and in the lifestyle of the population. Therefore, national authorities, the scientific community, and the food industry seek new formulations to facilitate these changes in an efficient way. Functional foods, together with a balanced diet, can offer improved health and the prevention of some illness [186]. In the case of incorporating RJ into food products, they would compensate or enhance the nutritional contribution of the diet that is acquired. It is an excellent remedy that can increase the contribution of nutrients of great importance for maintaining health. Currently, there are research works focused on new functional foods based on RJ, their properties, and the pharmacological effects on the human body [186].

It has been suggested that the inoculation of different quantities of RJ in skimmed milk has the potential to manage human diseases such as hyperglycemia (type 2 diabetes), hypertension, and several types of cancer, including breast and skin [174]. The study evaluated the beneficial properties of *Lactobacillus acidophilus* fermented milk with RJ. The biological parameters checked were total microorganisms, pH, antioxidant activity and inhibitory activities of angiotensin 1-converting enzyme, α-amylase, and two cancer cell lines growth, SW480 (colorectal) and MV3 (skin). Antioxidant activities increased after four hours of fermentation in skimmed milk fortified with RJ, and the accumulation of bioactive peptides with inhibitory activity of angiotensin 1-converting enzyme and with positive effects in the treatment of high blood pressure after one day was observed [174]. Rosenthal and colleagues evaluated the antibacterial effect of milk supplemented with RJ and found that this additive inhibits the growth of various Gram-positive and Gram-negative bacteria and mesophilic and thermophilic dairy starters in milk [175]. In addition, heat pasteurization did not inactivate the antibacterial activity of this additive. RJ, with a wide range of antimicrobial effects, would prevent the conversion of milk into fermented products.

#### 5.1.2. Supplements and Other Formulations

Different bioactive compounds previously described and mainly proteins, peptides, fatty acids, and phenolics give RJ multiple physiologic activities and medical applications. The beneficial effects as a result of the ingestion of RJ through capsules, tablets, or other preparations are based on experimental studies in which groups of patients were treated daily with different doses of this additive, whereas a control group was treated with a placebo.

RJ has been reported for its hypolipidemic beneficial effect, maintaining body weight and body fat [119]. The improvement of the dehydroepiandrosterone sulphate hormone concentration and the decrease in the serum total cholesterol and LDL-C levels after the administration of RJ in doses of 3.15 g/day for 12 weeks have been shown. Thus, RJ reduces the risk of cardiovascular disease without hepatic or renal damage. A recent study determining the effects of RJ on postmenopausal people (up to 60 years) indicated that women who received 1 g of RJ capsules for eight weeks had their menopausal symptoms significantly reduced [176]. The effect of the commercial dietary supplement (Memo^®^) on mental state evaluation in patients with moderate cognitive decline was also tested [177]. This product comprises 0.75 g of lyophilized RJ, 0.12 g of plant extracts *Ginkgo biloba*, and 0.15 g of *Panax ginseng*, and it was observed that ingesting one capsule once a day for 1 month on an empty stomach could be beneficial in dealing the cognitive decline typical of the aging process and of the early stages of the AD. However, more studies in this research field are needed to corroborate this benefit [177].

RJ has also demonstrated a positive effect against the premenstrual syndrome. Two months of consumption of one capsule daily (1 g RJ per capsule), starting on the first day of menstruation and continuing the same treatment over two consecutive menstrual cycles, alleviated premenstrual syndrome [178]. The nephroprotective effect of RJ was also tested. Cisplatin is one of the most effective antineoplastic drugs; nevertheless, the kidney damage it causes has limited its clinical use. In this sense, the role of RJ in the protection of cisplatin-induced acute nephrotoxicity in people with cancer was assessed, and RJ was found that to be involved in the nephroprotective effect against cisplatin toxicity [179]. In another experiment, the effect of JR in patients with dry eye affection was checked. A total of 43 Japanese volunteers with dry eye symptoms were subjected to six tablets of RJ daily (0.12 g/tablet) for two months. After that, the tear secretion volume, the meibum grade, and subjective dry eye symptoms, among others, were analyzed. The results indicate that the tear volume significantly increased after intervention in these patients [180].

The conclusion of another study revealed the improvement of mental health, erythropoiesis, and glucose tolerance after the intake of RJ. A total of 31 healthy adults were administered with three grams of this additive in 100 mL liquid/day for 6 months [181]. Doctors analyzed the effects and compared them with the control group. The initial results confirm modifications in anthropometric measurements and biochemical parameters from the beginning to 6 months after administration. Finally, the regular consumption of RJ demonstrated positive results on sperm and its mobility in infertility treatments [182].

### 5.2. Cosmetic Industry

RJ has been used since ancient times as an embellishing agent, including by historical figures such as Cleopatra. Nowadays, it is still very important in the cosmetic industry due to the rich content of bioactive compounds that confer diverse beauty and health benefits, on which care professionals focus their attention. Despite the previously described anti-inflammatory, antidiabetic, anti-lipidemic, antioxidant and antimicrobial properties, RJ is considered a natural anti-aging nutraceutical which leads to improved body composition and fertility enhancement [20].

Fatemeh Seyyedi and co-workers studied the therapeutic effects of vaginal cream of RJ on vaginal atrophy of postmenopausal women [183]. Participants were split into three groups. Women from the first group were administered with RJ vaginal cream 15%, the second group with vaginal Premarin commercial product, and the third group with placebo (lubricant), for three months. The results indicate that RJ has estrogen-like effects, since their cream was more effective than Premarin cream and lubricant in the improvement of quality of life in postmenopausal women. In the same way, the effectiveness of vaginal RJ in the treatment of sexual and urinary problems of postmenopausal women was confirmed in another experiment, leading to the conclusion that the effect is related to its estrogenic properties [185]. Another study revealed that 10-HDA may be useful in treating the dysfunction of the skin barrier. The activity of its synthetic counterpart, Hydroxydecine^®^, was evaluated and shown to be effective in restoring the skin barrier, reducing inflammation and hydrating dry skin [184].

## 6. Final Remarks and Conclusions

Royal jelly is a complex mixture of substances which is commonly utilized by the nutraceutical and cosmetic industry. Its composition is mainly formed by water, proteins, carbohydrates, lipids and, in a minor proportion, trace minerals, vitamins, and phenols. Among the proteins, MRJP and FAA are essential components of RJ, whereas, regarding lipids, 10-had is the most important substance, since it is a unique active compound. Phenolic and volatile compounds can be also important for their biological properties and their potential use as markers to differentiate RJ of different origins or harvesting times. RJ composition is highly variable, and so new analytical techniques are essential to study and address the authenticity and quality of the product. In respect to its biological properties, research focuses on anti-lipidemic, antioxidant, antimicrobial, anti-inflammatory and other effects, such as antiaging or estrogenic properties. However, further study of its mechanism of action is essential. RJ is consumed worldwide in different ways, its main use being as a functional food, although supplements have also been commercialized. The cosmetic industry has produced creams with estrogenic-like effects, but future lines of research are aimed at investigating RJ causing agents of antiaging effects. Due to this rapid expansion and increase in demand for RJ, the need to regulate the RJ market, currently dominated by China, becomes evident. These measures should not only be aimed at establishing check measures on the authenticity and origin of the RJ, but should also be aimed at preserving the environment and bees. Future research should focus on the full understanding of the routes of RJ substances to develop new applications and products for the nutraceutical and cosmetic, but also the pharmaceutical industry. In addition, it is essential to develop the LC-MS methodology, among other techniques, to study the entire composition of RJ.

## Figures and Tables

**Table 1:** Components of RJ related to their abundance and biological activities.

Compound	Molecular Group	% in Fresh RJ	Biological Activity	Reference
**Proteins**				
Proteins	-	9–18%	Stimulates proliferation of human monocytes and promotes proliferation of Jurkat lymphoid cell	[10,20,25,29,30]
MRJP1	Protein	5.89%	Nematicidal activity	[31]
			Antitumor effect	[7,32]
			Hypocholesterolemic effect	[15,33]
			Anti-hypertensive activity	[34]
			Allergen	[11,26]
Royalactin	Protein	0.25%	Increase in lifespan in invertebrates	[11,20]
			Stimulation of proliferation of rat hepatocytes	[35]
			Queen differentiation in honeybees	[35]
			Activation of a pathway that allows self-renewal of stem cells	[31]
MRJP2 and isoforms	Protein	1.41%	Antitumor effect	[7,30,32]
			Antimicrobial activity and Protection against oxidative stress	[36]
			Antibacterial activity	[37]
			Allergen	[11,26]
			Hepato-renal protective effect	[38]
			Antitumor effects	[39]
			Hepatocyte protection	[39]
			Wound-healing activity	[40]
MRJP3	Protein	1.66%	Modulation of immune responses of T cells	[30,41,42]
			Suppression of proinflammatory cytokine secretion.	[41]
			Immunomodulatory effect	[43]
			Wound-healing bioactivity	[40]
MRJP4	Protein	0.89%	Antimicrobial activity	[30,44]
MRJP5	Protein	0.64%	-	[30]
MRJP6	Protein	-	-	[30]
MRJP7	Protein	0.51%	Wound-healing bioactivity	[30,40]
MRJP8	Protein	-	-	[45]
MRJP9	Protein	-	-	[30]
Glucose oxidase	Enzyme	0.08%	Carbohydrate metabolism	[20,30]
			Antibacterial	[11,20,25,30]
Gluco-cerebrosidase	Enzyme	-	Hydrolysis	[46]
Alpha-glucosidase	Enzyme	-	Hydrolysis	[30,46]
Royalisin	Protein	0.83%	Antibacterial activity	[11,20,25,47,48,49]
			Antifungal activity	[11,20,47]
Apisimin	Peptide	0.13%	Stimulation of proliferation of human monocytes	[20,29,50]
Jelleines I-III	Peptide	0.37%	Antimicrobial activity	[1,20,25,49]
Jelleine IV	Peptide	-	-	[1,25,49]
Venom protein 2	Enzyme	-	Protection of larvae from diseases infection	[46]
Apolipophorin-III-like protein	Protein	0.08%	Antimicrobial	[20,25,51]
**Lipids**				
Lipids	-	3–8%	-	[10]
10-HDA	Fatty acid	0.75–3.39%	Antimicrobial activity	[17,20,52,53]
			Immunomodulatory activity	[54,55,56]
			Inhibitor of cancer growth	[57,58,59]
			Estrogenic activity	[58,60]
			Anti-inflammatory effect	[61]
			Activation of TRPA1 and TRPV1 receptors	[62]
			Increase longevity in *C. elegans*	[63]
			Neurogenesis inductor	[63]
			Protective effect against ultraviolet B in Human Skin	[64]
10-hydroxydecanoic acid (10-HDAA)	Fatty acid	0.78–1.05%	Estrogenic activity	[58,59,60]
			Activation of TRPA1 and TRPV1 receptors	[62]
8-hydroxy octanoic acid	Fatty acid	0.18–0.39%	Varroa-repellent activity	[59,65]
3-hydroxydecanoic acid	Fatty acid	0.05–0.09%	Antifungal activity	[59,66]
3,10-dihydroxydecanoic acid	Fatty acid	0.26–0.46%	Immunomodulatory activity: Stimulation of dendritic cell differentiation	[55,59,67]
9-hydroxy-2-decenoic acid	Fatty acid	0.07–0.15%	Signal components (pheromone) of honeybee queen	[14,59]
1,10-decanedioic acid (sebacic)	Fatty acid	0.15–0.24%	Estrogenic activity	[58,59,60]
		-	Anti-inflammatory effect	[61]
2-Decenedioic	Fatty acid	0.18–0.33%	-	[59]
Phenols	Lipid	0.24–0.6%	Antioxidant activity	[59,62,68]
Ferulic acid	Phenol	12.95–18.93 µg/kg	Antioxidant activity	[59,69]
Waxes	Lipid	0.3–0.36%	-	[62]
Steroids	Lipid	0.18–0.24%	Effects on collagen synthesis	[59,62]
24-methylene cholesterol	Steroid	6.06 mg/lipid	Estrogenic activity	[27,60,70]
Phospholipids	Lipid	0.02–0.04	-	[62]
**Vitamins**				
Vitamin A	Vitamin	1.10 mg/100 g	Immunity, maintenance of the visual system, and maintenance of epithelial cellular integrity	[71,72]
Vitamin B1	Vitamin	2.06 mg/100 g	Transketolation, metabolism of fats, proteins and nucleic acids	[71]
Vitamin B2	Vitamin	2.77 mg/100 g	Precursor of FMN and FAD	[71]
Niacin (B3)	Vitamin	42.42 mg/100 g	Increase HDL cholesterol levels	[71]
Vitamin B5 (Pantothenic acid)	Vitamin	52.80 mg/100 g	Constituent of coenzyme A, fatty acid metabolism	[71,73]
Vitamin B6	Vitamin	11.90 mg/100 g	Transamination and decarboxylation of amino acids	[71]
Vitamin B9 (Folic acid)	Vitamin	0.40 mg/100 g	DNA biosynthesis and methylation	[74]
Vitamin B12	Vitamin	0.15 mg/100 g	Formation of red blood cells and maintenance of the central nervous system.	[71]
Vitamin C (Ascorbic acid)	Vitamin	2.00 mg/100 g	Antioxidant	[71]
Vitamin D	Vitamin	0.2 mg/100 g	Calcium absorption	[74]
Vitamin E	Vitamin	5.00 mg/100 g	Antioxidant activity	[71]

**Table 2:** Antimicrobial activity of RJ compounds in terms of minimal inhibitory concentration (MIC) (µg/mL). Based on [1,48,51,138,143,144,145,146,147].

	*A*	*B*	*C*	*D*	*E*	*F*	*G*	*H*	*I*	*J*	*K*	*L*	*M*	*N*	*O*
*Candida albicans*		1450	1250	700	1320	2.5	2.5–10		1100	0.125–1	1340	1250		200	1100
*Candida glabatra*		1170	1170	350					850		800	980		150	1250
*Candida tropicalis*		1120	900	490					900		970	950		180	950
*Paenibacillus larvae*	0.6–2.8						40						6		
*Vibrio parahaemolyticus*													4		
*Enterobacter cloacae*		1630	770	570	1500	10	15		1500		1330	1200		1100	900
*Escherichia coli*		1150	400	1100	1180	2.5	10–15	15	1100	10	980	950		1450	1500
*Lactobacillus acidophilus* and *Lactobacillus helveticus*										10					
*Pseudomonas aeruginosa*		1120	650	640	1180	10	15–80	30	750		980	940	10	900	670
*Staphylococcus aureus*		670	550	350	350	10	15–200	30	450		700	670	7–250	950	990
*Staphylococcus epidermidis*		990	400	250	400		200		780		720	740		880	950
*Staphylococcus intermedius* and *Staphylococcus xylosus*													4–12		
*Staphylococcus saprophyticus*						15		30							
*Streptococcus mutans*		1370	780	350	670				980		800	720		1140	780
*Streptococcus viridans*		900	840	180	780				800		700	580		950	740
*Streptococcus alactolyticus*													9		
*Bacillus subtilis*						10	30–40						60		
*Klebsiella pneumoniae*		1250	1450	470		10	15		900		1260	980		1350	1640
*Listeria monocytogenes*							200								
*Salmonella enterica Paratyphi*							200								
*Salmonella cholearasuis*													9		
*Salmonella (infantis, typhi-murium)*										10					
*Clostridium tetani*													250		
*Micrococcus luteus (Sarcina lutea)*													125		
*Bifidobacterium (adolescentis, bifidum, breve, infantis, lon-gum)*										10					

A: 10-HDA; B: 3-(4-hydroxy-3-methoxyphenyl)-propionic acid; C: 3,10-dihydroxy-decanoic acid; D: 3-hydroxy-dodecanedioic acid; E: 9-hydroxy-2-nonanone; F: Jelleine-I; G: Jelleine-II; H: Jelleine-III; I: Methyl paraben; J: Royal Jelly; K: RJ(CH2CL2ex-tract); L: RJ(MeOHextract); M: Royalisin; N: Sebacic acid; O: Trans-10-hydrixydec-2-enoic acid.

**Table 3:** Royal Jelly industry applications and their benefits to promote human health.

Applications	Administration of RJ/Dose in Humans	Health Benefits	Ref.
**NUTRACEUTICAL INDUSTRY**			
*Functional foods*			
Skimmed milk fortified with RJ (Probiotics)	*Lactobacillus acidophilus* fermented milk with RJ	Increase in antioxidant activities. Accumulation of bioactive peptides with antihypertensive effect (inhibitory activity of angiotensin 1-converting enzyme).	[174]
Milk supplemented with RJ		Inhibits some Gram-positive and Gram-negative bacteria growth and mesophilic and thermophilic dairy starters. Prevention of the conversion of milk into fermented products	[175]
*Supplements/industry formulations*			
Capsules (1 g RJ/capsule)	Daily intake of oral 1 capsule for 8 weeks	Effectiveness in alleviating the menopausal symptoms.	[176]
Capsules (0.35 g RJ/capsule)	Consumption of 9 capsules per day for 3 months	Significantly reduction in the serum total cholesterol and low-density lipoprotein cholesterol levels.	[119]
Memo^®^ Capsules (0.75 g of lyophilized RJ/capsule)	One Memo^®^ capsule before breakfast daily for 4 weeks.	Cognitive impairment treatment representative of the early phases of Alzheimer’s disease (more studies needed).	[177]
Capsules (1 g RJ/capsule)	Two months intake of one RJ capsule daily	Effectiveness in reducing the premenstrual syndrome	[178]
Capsules (quantity not found).	Two capsules of RJ	Nephroprotective effect against cisplatin toxicity in cancer assisted for cisplatin chemotherapy.	[179]
Tablets (0.12 g RJ/tablet)	Ingestion of 6 tables daily for 8 weeks	Increased tear volume in patients with dry eye	[180]
Liquid formulation	Six-month intake of RJ (3 g of RJ in 100 mL liquid/day)	Improvement of erythropoiesis, glucose tolerance and mental health.	[181]
		Positive results on sperms and its mobility in infertility treatments	[182]
**COSMETIC INDUSTRY**			
Cream	RJ vaginal cream 15%	Estrogen-like effects	[183]
Cream	Cream with 10-Hydroxy-2-decenoic fatty acid exclusive of RJ.	Possible treatment in the dysfunction of the skin barrier	[184]
Cream	Vaginal cream of RJ 15% for 3 months	Estrogenic properties to treat sexual and urinary problems in women	[185]

## Data Availability

No new data were created or analyzed in this study. Data sharing is not applicable to this article.

## Abbreviations

RJ	Royal Jelly
10-HDA	10-hydroxy-2-decenoic acid
MRJP	Major Royal Jelly Proteins
FAA	Free Amino Acids
HDAA	10-hydroxydecanoic acid
VC	Volatile Compounds
HDL-C	High-Density Lipoprotein Cholesterol
LDL-C	Low-Density Lipoprotein Cholesterol
ROS	Reactive Oxygen Species
NAFLD	Nonalcoholic Fatty Liver Disease
RNS	Reactive Nitrogen Species
AMPs	Antimicrobial Peptides Appear
AD	Alzheimer’s Disease
HS-SPME/GC–MS	Headspace Solid-Phase Microextraction/Gas Chromatography-Mass Spectrometry
DPPH	2,2-diphenyl-1-picrylhydrazyl
RA	Rheumatoid Arthritis
VEGF	Vascular Endothelial Growth Factor
pRJ	protease-treated RJ
